# Clinical Characteristics of Plantar Warts and Association With BMI: A Retrospective Case Series Study

**DOI:** 10.1111/srt.70303

**Published:** 2025-12-12

**Authors:** Zhao Li, Yangyang Ma, Ke Bian, Xiaowen Li, Jifeng Liu, Ping Wang

**Affiliations:** ^1^ Department of Dermatology Third People's Hospital of Hangzhou Hangzhou 310009 Zhejiang People's Republic of China

**Keywords:** body mass index (BMI), clinical characteristics, correlation, dermatoscopy, plantar warts

## Abstract

**Background:**

Plantar warts are among the most common skin lesions caused by the human papillomavirus (HPV) and are highly resistant to treatment. Obesity is a well‐established factor for skin disease, yet few studies have focused on its association with plantar warts. This study aimed to describe the clinical characteristics of plantar warts and their relationship with body mass index (BMI).

**Methods:**

This retrospective case series included patients with plantar warts treated at the dermatology department of the Third People's Hospital of Hangzhou between May 2023 and March 2025. An electronic medical record system was used to collect participants’ demographic and clinical data. Dermoscopy was used to evaluate wart dermatological features, focusing on two key aspects: vascularity and surface patterns. Patients were categorized into two groups based on BMI: BMI ≥ 24.0 kg/m^2^ and BMI < 24.0 kg/m^2^, to compare their dermatological characteristics.

**Results:**

A total of 262 patients with plantar warts were included in the study (average age: 37 ± 12.75 years), with more females (55.73%) than males (44.27%). Most plantar warts were single lesions (66%) and appeared predominantly on the soles (49%) and toes (29%). Dermoscopically, the majority exhibited dotted blood vessels (79%) and a yellow or brown color, both for the background (74%) and surface pattern (68%). Among the 262 patients, 90 were in the BMI ≥ 24.0 kg/m^2^ group (34.25%), and 172 were in the BMI < 24.0 kg/m^2^ group (65.65%). Compared to the BMI< 24 kg/m^2^ group, the BMI≥24 kg/m^2^ group had a significantly higher proportion of males (61.11% vs. 35.47%, *p *< 0.001), frogspawn (35.56% vs. 19.77%, *p* = 0.012), and papilliform patterns (7.78% vs. 5.81%). No statistically significant differences were observed in age, number of warts, location, background color, and vascular characteristics between the two groups (*p *> 0.05).

**Conclusions:**

This case series demonstrate the utility of dermoscopy as a non‐invasive and cost‐effective tool for identifying and characterizing the features of plantar warts. Our descriptive findings revealed variations in the dermoscopic patterns of plantar warts across different BMI categories. Our findings offer preliminary insights into the clinical presentation of plantar warts, paving the way for future studies with more robust design and statistical analysis.

## 1 Introduction

Plantar warts, benign proliferative cutaneous lesions of the foot caused by human papillomavirus (HPV) infection, are among the most common diseases encountered in dermatologic practice worldwide [1]. It is estimated that HPV affects approximately 40% of the population, among whom 7%–12% eventually develop plantar warts [2]. Moreover, the annual incidence of plantar warts in the general population is estimated to be 14% [2]. Plantar wart incidence is influenced by various factors, including age, sex, race, and health status, as well as geographic, seasonal, behavioral, and socioeconomic factors [2, 3]. Children and adolescents are more commonly affected due to their developing immune systems and increased exposure to public spaces, with peak incidence between 12 and 16 years of age [4]. Women tend to have higher rates of plantar warts than men throughout their lifetime [2]. Additionally, plantar warts occur more frequently in individuals of certain ethnicities, those with weakened immune systems, and those living in certain geographic areas [2, 3].

Plantar warts typically present as yellowish or brownish‐yellow callosities or flat papules with rough surfaces distinct boundaries and are often surrounded by a slightly elevated horny ring [5]. Located on the soles of the feet, plantar warts commonly cause pain or the sensation of a stone or swelling under the foot, which is often experienced during walking or standing [2]. Plantar warts not only impair patients' standing and walking abilities, leading to pain and discomfort, but also impact aesthetics, potentially detrimentally affecting patients' mental well‐being and overall quality of life [6]. Traditional diagnosis of plantar warts is limited to visual assessment or cultures, which can be slow and less sensitive [7]. Molecular diagnostics, such as polymerase chain reaction (PCR) tests, provide a highly sensitive and specific method for diagnosing plantar warts, complementing traditional methods [7]. PCR's superior sensitivity allows it to detect even trace amounts of a pathogen's genetic material, enabling earlier and more accurate diagnosis [8]. For example, a study by Wei et al. [9] demonstrated a 99% specificity of real‐time PCR for detecting HPV in skin tissue, reinforcing its value in confirming challenging cases [9]. However, this advanced technology comes with its own set of challenges, including high expenses, invasive procedures, and the risk of inaccurate results due to contamination or non‐specific amplification [8, 10]. Dermoscopy provides a non‐invasive method to bridge the gap between macroscopic and microscopic examination, with studies showing a sensitivity of 100% [10]. Currently, multiple treatment modalities exist for plantar warts, encompassing systemic approaches, local pharmacological interventions (e.g., salicylic acid, imiquimod), physical modalities (e.g., cryotherapy, laser therapy), photodynamic therapy, and surgical excision. However, no universal therapy yields consistently favorable outcomes for all patients, with a notably high recurrence rate [11]. Therefore, identifying modifiable risk factors for plantar warts is crucial in informing targeted and effective prevention and intervention efforts.

Previous research has consistently shown a significantly positive association between obesity and the susceptibility to and severity of various skin conditions [12, 13]. For instance, a systematic review and meta‐analysis identified 104 variables across 51 studies, which concluded that higher body mass index (BMI) was the only significant factor associated with plantar fasciopathy [12]. Obesity is a significant global health concern, affecting over 1 million people in the global population [14]. In China, approximately half of adults are overweight (34.3%) or obese (16.4%) [15]. Excess weight can cause increased pressure on the feet, which may further contribute to the inward growth and potential discomfort of plantar warts [13]. Additionally, obesity significantly impacts the local skin microenvironment of the feet through a complex interplay of physiological changes [16]. These changes include modified foot structure and mechanics, heightened local mechanical stress, increased secretion of pro‐inflammatory mediators, and compromised skin microcirculation [16]. Collectively, these factors contribute to an altered local skin microenvironment in the feet of obese individuals, making them more susceptible to skin problems and infections [16].

However, few studies have focused exclusively on plantar warts and their association with body weight, representing a significant research gap. The high prevalence of plantar warts and their association with obesity indicate the need for more clinical evidence to elaborate on the characteristics and underlying mechanisms of plantar warts. Therefore, this study aimed to describe the clinical characteristics of plantar warts and compare them between those with high and lower body mass index (BMI). Our study would offer significant insights into the prevention and management of plantar warts in the general population, especially those with obesity.

## 2 Methods

### 2.1 Study Design and Participants

This investigation was designed as a case series to provide a descriptive account of patients presenting with plantar warts. As such, the analysis focuses on summarizing the key characteristics and outcomes of this patient cohort rather than conducting inferential statistical analysis. The study was conducted at the Department of Dermatology in the Third People's Hospital of Hangzhou City from May 2023 to March 2025. Participants were patients diagnosed with plantar warts based on established diagnostic criteria [17], characterized by flat or round papules, plaques with keratosis, rough and uneven surfaces, distinct boundaries, and encircled by a keratinous ring. Inclusion criteria were as follows: (1) meeting the aforementioned diagnostic standards for plantar warts, (2) aged between 18 and 65 years, and (3) with initial dermatological images available. Exclusion criteria were as follows: (1) recent treatments, such as laser therapy, cryotherapy, or topical corrosive agents; (2) cases of incomplete wart removal; and (3) pregnant or lactating women. As a retrospective study, this study met the ethical standards for exemption from informed consent and was approved by the Ethical Committee of the Third People's Hospital of Hangzhou City (Ethics No. [2025KA173]).

### 2.2 Data Collection

An electronic medical record system was used to collect participants’ demographic and clinical data, including sex, age, height, weight, and details regarding lesion location and quantity. Dermoscopy was used to evaluate wart dermatological features, focusing on two key aspects: vascularity and surface patterns. Vascularity is classified based on the types of vessels observed, such as dots and linear vessels. The surface scales are classified based on their appearance, including yellow structureless, frogspawn, and papilliform patterns. Frogspawns refer to the appearance of tiny black or red dots surrounded by white halos when viewed under a dermatoscope. These dots are clotted capillaries (blood vessels) inside the wart, which are a key diagnostic feature distinguishing warts from other skin conditions like corns. Papilliform patterns refer to the rough, bumpy, or finger‐like projections that form a warty surface. Under a dermoscope, these warts show a distinct, warty pattern with a yellowish surface and interrupted skin lines, often with tiny black dots of clotted capillaries. Other dermoscopic characteristics included background color (red/pink or yellow/brown).

### 2.3 Statistical Analysis

All statistical analyses were performed using SPSS 26.0. Normally distributed measurement data are presented as mean ± standard deviation (*x* ± *s*), while categorical data are presented as frequency and percentage (*n*, %). Patients were categorized into two groups based on the Chinese adult body mass index standard [18]: BMI ≥ 24.0 kg/m^2^ and BMI < 24.0 kg/m^2^. The chi‐square test was used to compare the demographic and clinical characteristics between the two groups. In cases of insufficient theoretical frequencies, the Fisher exact probability method was employed. The significance level of *p* < 0.05 was used to determine statistical significance.

## 3 Results

### 3.1 Sample Characteristics

**FIGURE 1:**
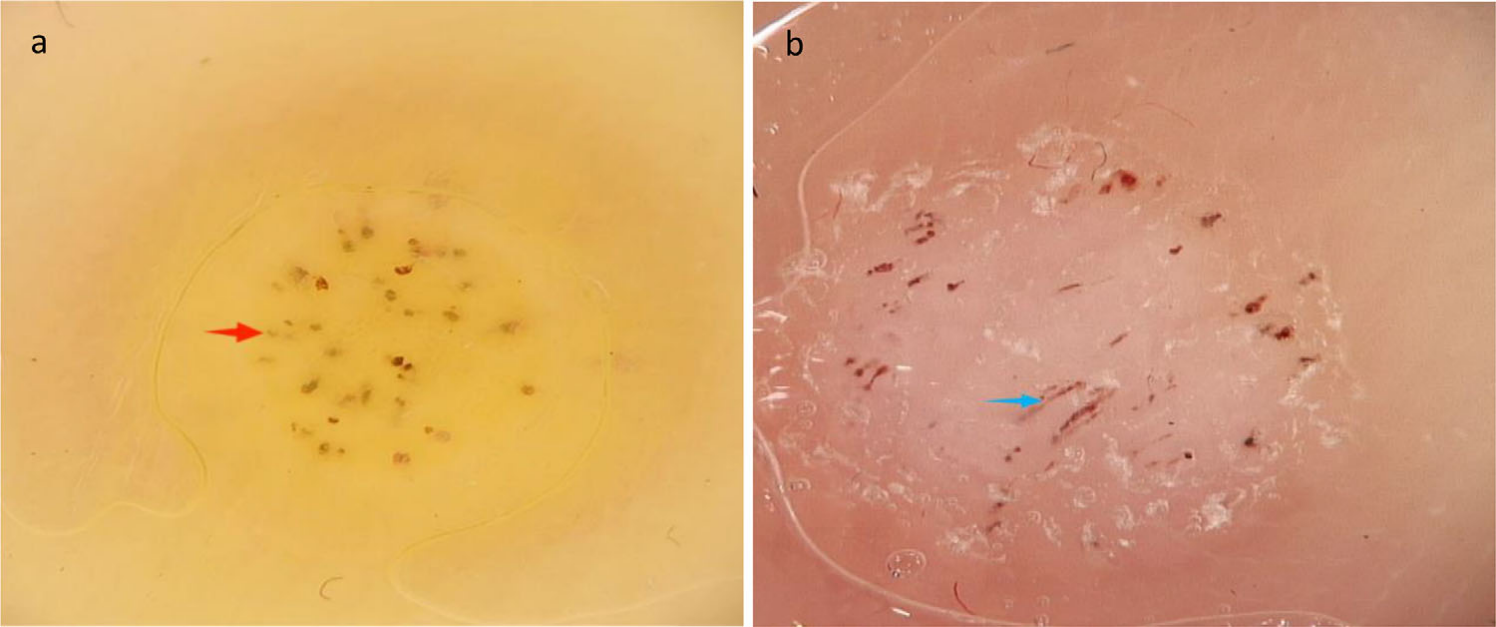
Dermoscopic evaluation criteria for the vascular distribution and background color of plantar warts: (a) yellow background with petechial hemorrhage (red arrow), (b) pink background with linear hemorrhage (blue arrow).

**FIGURE 2:**
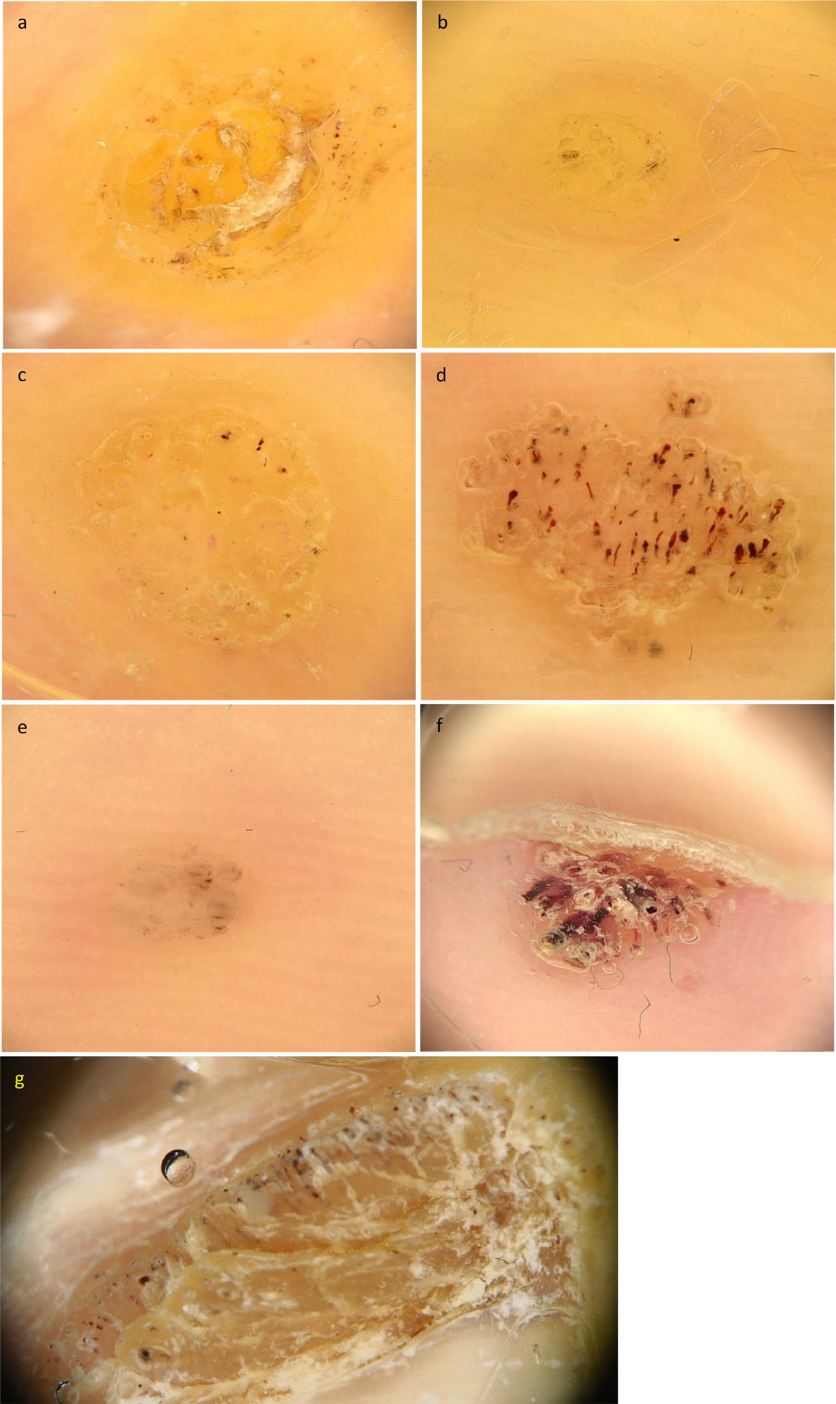
Dermoscopic evaluation criteria for the surface scales of plantar warts: Surface patterns: (a and b) yellow structureless, (c–e) frogspawn, (f and g) papilliform pattern.

Our final analysis included 262 patients diagnosed with plantar warts. The demographic and clinical characteristics of the total sample are summarized in Table 1. Participants had an average age of 37±12.75 years, with the age group of 25–34 years accounting for the largest proportion (34.35%). There were slightly more females (*n* = 146, 55.73%) than males (*n* = 116, 44.27%). Regarding dermatological characteristics, most had a single occurrence (66.03%), with a vascularity of dotted blood vessels (78.63%), a surface pattern of yellow structureless area (68.32%), and a background color of yellow or brown (74.43%). As for location, soles account for the largest proportion (49.24%), followed by toes (29.01%). Details of dermatological findings are presented in Figures 1 and 2.

**TABLE 1:** Demographic and clinical characteristics of the total sample (*n* = 262).

Variables	Category	*n* (%)
Age (years)	18—24	44 (16.79%)
	25—34	90 (34.35%)
	35—44	67 (25.57%)
	45—54	29 (11.07%)
	55—65	32 (12.21%)
Sex	Male	116 (44.27%)
	Female	146 (55.73%)
Number of warts	Single	173 (66.03%)
	Multiple	89 (33.97%)
Location	Toes	76 (29.01%)
	Sole	129 (49.24%)
	Metatarsal arch area	8 (3.05%)
	Lateral area	8 (3.05%)
	Heel	41 (15.65%)
Vascularity	Dotted blood vessels	206 (78.63%)
	Linear blood vessels	56 (21.37%)
Surface patterns	Yellow structureless area	179 (68.32%)
	Frogspawn	66 (25.19%)
	Papilliform	17 (6.49%)
Background color	Red or pink	67 (25.57%)
	Yellow or brown	195 (74.43%)

### 3.2 Comparisons Between the Two BMI Groups

Among the 262 patients, 90 were in the BMI ≥ 24.0 kg/m^2^group (34.25%), and 172 were in the BMI < 24.0 kg/m^2^group (65.65%). Table 2 shows the comparison of the demographic and clinical characteristics between the BMI < 24.0 kg/m^2^group and the BMI ≥ 24.0 kg/m^2^group. There were significant differences in gender and surface pattern between the two groups. The BMI ≥ 24 kg/m^2^ group had a significantly higher proportion of males than the BMI < 24 kg/m^2^ group (61.11% vs. 35.47%, *p *< 0.001). Regarding the surface pattern of lesions, the BMI < 24 kg/m^2^ group had a significantly higher proportion of yellow structureless area (74.42% vs. 56.67%); while the BMI ≥ 24 kg/m^2^ group had significantly higher proportions of frogspawn (35.56% vs. 19.77%) and papilliform pattern (7.78% vs. 5.81%) (χ^2^ = 8.923, *p* = 0.012). No statistically significant differences were observed in age, number of warts, location, background color, and vascular characteristics between the two groups (*p *> 0.05).

**TABLE 2:** Comparison of demographic and clinical characteristics between the two BMI groups (*n* = 262).

Variables	Category	BMI < 24 kg/m^2^ (*n* = 172)	BMI ≥ 24 kg/m^2^ (*n* = 90)	*X* ^2^	*p*
Age (years)	18–24	31(18.02%)	13 (12.62%)	3.652	0.455
	25–34	63(36.63%)	27 (26.21%)		
	35–44	40(23.26%)	27 (26.21%)		
	45–54	20(11.63%)	9 (8.74%)		
	55–65	18(10.47%)	14 (13.59%)		
Sex	Male	61(35.47%)	55 (61.11%)	15.571	< 0.001
	Female	111 (64.53%)	35 (38.89%)		
Number of warts	Single	115 (66.86%)	58 (64.44%)	0.154	0.695
	Multiple	57 (33.14%)	32 (35.56%)		
Location	Toes	55 (31.98%)	21 (20.39%)	6.508	0.164
	Sole	79 (45.93%)	50 (48.54%)		
	Metatarsal arch area	5 (2.91%)	3 (2.91%)		
	Lateral area	3 (1.74%)	5 (4.85%)		
	Heel	30 (17.44%)	11 (10.68%)		
Vascularity	Dotted blood vessels	140(81.40%)	66 (73.33%)	2.285	0.131
	Linear blood vessels	32(18.60%)	24 (26.67%)		
Surface patterns	Yellow structureless area	128 (74.42%)	51 (56.67%)	8.923	0.012
	Frogspawn	34 (19.77%)	32 (35.56%)		
	Papilliform	10 (5.81%)	7 (7.78%)		
Background color	Red or pink	39(22.67%)	28(31.11%)	2.210	0.137
	Yellow or brown	133(77.33%)	62(68.89%)		

## 4 Discussion

### 4.1 Summary of the Findings

Plantar warts are benign growths that develop on the soles of the feet, resulting from HPV infection, typically found on weight‐bearing areas such as the heel, metatarsal head, and interdigital spaces [19]. Among over 200 HPV subtypes, the most prevalent ones affecting the foot include types 1, 2, 4, 10, 27, and 57 [17, 20]. Plantar warts are contagious, common, and resistant to treatment, negatively affecting the quality of life and general well‐being of the affected individuals. In this study, we comprehensively described the characteristics of patients with plantar warts and explored their association with BMI. Our descriptive findings reveal variations in wart presentation across the different BMI categories, with more frogspawn and papilliform surface patterns observed in the BMI≥24.0 kg/m^2^group, though no causal relationship can be inferred.

### 4.2 Demographic and Dermatological Features of Plantar Warts

Our study revealed gender and age disparities in the epidevvmiology of plantar warts. The age group of 25–34 years had the largest proportion of the total sample, which was consistent with the literature showing a peak incidence at 27.8 years [21].In addition, this study noted a higher prevalence of plantar warts in adult women compared to men, also aligning with the bulk of previous studies showing that females have a greater lifetime risk of plantar warts than males [22]. This may be explained by the contemporary footwear preferences, heightened exposure in communal settings (e.g., swimming pools, yoga studios), and fluctuations in estrogen levels in females, leading to a higher susceptibility to plantar warts.

Using dermoscopy, we identified several predominant dermatological features of plantar warts, including single occurrence, dotted blood vessels, a yellow structureless area, and a background color of yellow or brown. Dermoscopy, a non‐invasive and cost‐effective diagnostic tool, is highly useful in identifying characteristic vascular patterns and surface features of plantar warts, earning it the moniker “dermatologist's stethoscope” [23]. Dermatoscopy enables clear visualization of characteristic wart structures, including vascular patterns, keratinized features, and background color attributes, which not only enhances diagnostic precision but also furnishes an objective framework for evaluating lesion activity and severity [24]. Studies suggest that dermoscopy has a similar diagnostic performance to that of PCR, indicating its cost‐effectiveness and clinical utility in the diagnosis and treatment of plantar warts [10].

### 4.3 BMI and Plantar Warts

Body mass index (BMI) is a crucial health indicator, and the prevalence of high BMI (overweight and obesity) has been escalating globally [25]. China, with its large population, faces a considerable burden of high BMI, with a rising prevalence of both adult and adolescent obesity [26]. High BMI can contribute to a variety of skin problems due to changes in skin structure, function, immune response, and increased susceptibility to infections. Skin manifestations linked to obesity encompass acanthosis nigricans, follicular keratosis, dermatophytes, palmoplantar keratosis, and skin infections [27]. Excess weight associated with overweight/obesity alters foot anatomy, increasing pressure on plantar weight‐bearing regions (e.g., heel and metatarsal area) and causing plantar hyperkeratosis [28]. The redistribution of excessive weight to bony prominences can further induce local recurrent mechanical trauma and microinjuries [28]. Consequently, this creates a gateway for HPV infiltration, heightening the infection risk.

The preliminary comparison between the two BMI groups showed no significant differences across several key characteristics, including age, number of warts, location, vascularity, and background color. However, the BMI ≥ 24.0 kg/m^2^group had more male patients than the BMI < 24.0 kg/m^2^group, possibly influenced by male immune responses, behavioral patterns, occupational practices, and hygiene conditions. In addition, the BMI ≥ 24.0 kg/m^2^group also had

Higher proportions of frogspawn and papilliform patterns for plantar warts. This finding challenges the previous belief that obesity merely results in localized protective hyperkeratosis [24]. A high BMI can lead to both increased mechanical stress on the sole and alterations in the skin microenvironment, including chronic inflammation and immune dysfunction. This, in turn, may facilitate HPV replication and persistent infection, exacerbate abnormal keratinocyte proliferation, and induce alterations in vascular distribution and morphology. The interplay of these diverse mechanisms renders plantar warts in overweight or obese individuals a more intricate dermatological condition. While this observation is intriguing, our study's retrospective, single‐center, and descriptive design does not allow us to establish a causal relationship or to infer statistical significance. The observed differences could be influenced by a number of factors not controlled for in this case series, and this descriptive finding should be viewed as preliminary.

### 4.4 Limitations

This study has several limitations. A major limitation of this study is its design as a case series, which precludes the use of multivariate analysis to account for confounding factors. As such, we could not statistically adjust for the potential influence of pre‐existing conditions, such as hyperkeratosis or helomas, on the observed outcomes. While our preliminary comparisons did not indicate significant differences between BMI groups on several key characteristics, this analysis was descriptive in nature. Future studies employing a controlled design with multivariate statistical analyses are needed to more precisely evaluate the impact of potential confounding variables on patient outcomes. Another limitation is the absence of molecular confirmation for HPV infection. As a case series, our diagnostic approach relied on established clinical and dermoscopic criteria. While dermoscopy has demonstrated high sensitivity in diagnosing warts, it is not the gold standard for confirming an underlying HPV infection. As such, we cannot exclude the possibility that some lesions included in our study were not verrucae but rather hyperkeratotic lesions of a different etiology, which may have altered our results. Future studies incorporating molecular techniques like PCR testing would be valuable for providing definitive diagnostic confirmation and reducing potential sources of misclassification bias. Furthermore, the findings of this study may be limited in their generalizability, as it was conducted at a single center using a retrospective design that relied on pre‐existing medical records. Future multicenter, prospective studies with standardized data collection methods and a more robust design are warranted to confirm these preliminary findings and to address the limitations of the current study.

### 4.5 Clinical Implications

While this case series is descriptive and does not allow for causal inferences or the statistical evaluation of confounding variables, our observations provide valuable clinical insights. The detailed characterization of dermoscopic patterns can aid clinicians in the non‐invasive diagnosis of plantar warts. By systematically documenting and classifying these patterns, our study offers a valuable reference for clinicians, especially those in settings where more definitive diagnostic tools like PCR are not readily available or are too expensive. For instance, the consistent identification of dermoscopic hallmarks, such as the interruption of skin lines and the presence of thrombosed capillaries (appearing as red or black dots), is a key differentiator from other common foot lesions like calluses and corns. This helps clinicians avoid misdiagnosis and unnecessary treatments. The descriptive data on the variety of patterns observed also helps manage patient expectations, as it illustrates the variability in the presentation of plantar warts.

As a retrospective case series, our study serves as a hypothesis‐generating foundation for more robust investigations. The patterns observed, including the descriptive differences across BMI groups, could be explored in larger, prospective, multicenter studies. Such future research could incorporate molecular confirmation through PCR to definitively link dermoscopic patterns with specific HPV subtypes and to exclude other forms of hyperkeratosis. Furthermore, future studies could perform multivariate analyses to formally test for associations between dermoscopic patterns, BMI, and other patient characteristics. This would help identify potential prognostic markers or treatment response predictors. The detailed descriptive data collected in this study can inform the development of standardized protocols for dermoscopic examination and the design of subsequent controlled trials. Ultimately, our work provides a necessary starting point for more rigorous research aimed at improving the diagnostic accuracy and clinical management of plantar warts.

## 5 Conclusions

In summary, this descriptive case series identified several predominant dermoscopic features of plantar warts. This case series demonstrates the utility of dermoscopy as a non‐invasive and cost‐effective tool for identifying and characterizing the features of plantar warts. We also observed distinct dermatological patterns in plantar warts among patients across varying BMI categories, including higher proportions of frogspawn and papilliform patterns in the BMI ≥ 24.0 kg/m^2^ group. While our descriptive findings cannot account for confounding variables, they offer preliminary insights into the clinical presentation of plantar warts. Future research, incorporating molecular confirmation via PCR and appropriate statistical analyses, could explore potential associations between patient characteristics and dermoscopic patterns more robustly. Ultimately, this would facilitate a more comprehensive understanding of plantar warts and could inform the development of more tailored and targeted therapeutic approaches for patients in the future.

## Funding

This work was supported by Hangzhou City Science and Technology Plan Guidance Project (20211231Y032)

## Conflicts of Interest

The authors declared no conflicts of interest.

## Data Availability

The data that support the findings of this study are available from the corresponding author upon reasonable request.
